# Evaluating the Connection Between Rural Travel Time and Health: A Cross-Sectional Analysis of Older Adults Living in the Northeast United States

**DOI:** 10.1177/21501319241266114

**Published:** 2024-07-25

**Authors:** Madison Hearn, Casey Pinto, Jennifer L. Moss

**Affiliations:** 1 Penn State College of Medicine, Hershey, PA, USA

**Keywords:** rural, travel time, healthcare access, health outcomes, geography

## Abstract

**Introduction:**

To characterize the impact of rural patients’ travel time to obtain healthcare on their reported utilization of preventive healthcare services and personal health outcomes.

**Methods:**

Online survey data from rural adults ages 50+ years living in the Northeastern United States were collected from February to August 2021. Study measures included self-reported travel time to obtain healthcare, use of preventive healthcare, and health outcomes. The associations between travel time with use of preventive care and health outcomes were assessed using linear, Poisson, and logistic regression analyses controlling for demographic variables.

**Results:**

Our study population included 1052 rural adults, with a mean travel time of 18.5 min (range: 0-60). Travel time was greater for racial/ethnic minority participants and for higher-income participants (both *P* < .05), but it was not associated with use of preventive healthcare. Greater travel time was associated with poorer mental health and more comorbidities, including cancer and diabetes (all *P* < .05).

**Conclusions:**

Travel time varied by patient demographic factors, and it was associated with mental health and comorbidities. There was no association between travel time and preventive care use, suggesting that other barriers likely contribute to suboptimal use of these services within rural communities. Further research is needed to elucidate the causal pathways linking travel time to mental health and comorbidities within rural communities, as increased travel may exacerbate intrarural health disparities.

## Introduction

The approximately 15% of Americans living in rural areas experience healthcare disparities including lower utilization of preventive care,^1^ higher levels of chronic disease,^2^ and higher mortality^3^ when compared to urban populations. Significant barriers to accessing healthcare exist in rural communities, including geographical access to healthcare. Travel time is a barrier to healthcare access that disproportionately impacts rural areas, with rural patients travelling 2 to 3 times farther to obtain healthcare than their urban counterparts.^4^ Prior research has established that rural patients travel the farthest for specialty care, especially surgical and cancer care.^4,5^ Increasing community hospital closures in the United States over the past decade have resulted in a centralization of services in larger and more urban communities, which has likely exacerbated this problem.^6,7^

Some studies suggest that increased travel time may also be associated with decreased use of preventive healthcare and poorer health outcomes^8^; however, contradictory evidence exists.^9^ A survey of rural residents in North Carolina found that distance traveled to regular care was negatively associated with regular checkups and chronic illness care.^8^ Similarly, a systematic review identified that the majority of studies in Westernized countries reported worse health outcomes for patients who lived farther away from healthcare facilities, although some studies reported no relationship or the opposite relationship.^9^ These inconsistent findings may be related to the focus on rural/urban differences in distance to care and the lack of consideration of the heterogeneity that exists within and across rural communities. Experts recommend moving beyond identifying rural/urban differences and considering within-group differences to better understand rural health disparities.^10,11^ To date, intrarural differences, including differences in travel time to healthcare, have been poorly characterized.

The purpose of this study was to identify the sociodemographic variables associated with travel time to healthcare, and potential effects of travel time on health, among rural older adults in the Northeastern region of the United States. Specifically, we aimed to characterize the association between travel time and use of preventive healthcare and health outcomes among rural older adults (ages 50+ years). This study will help to address debate in the literature regarding the relationship between travel time and rural health, which may help to inform policies that advance healthcare access and health equity for this population.

## Methods

**Figure 1.**
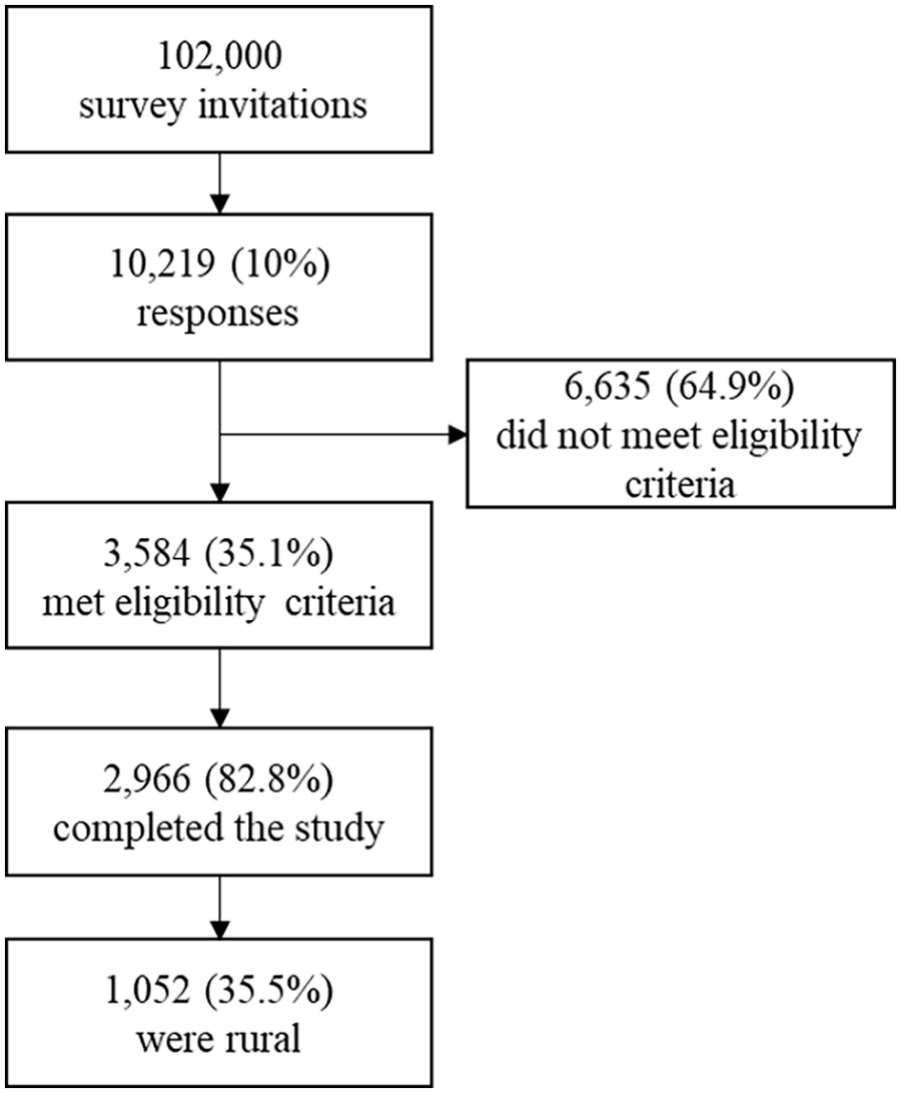
Flow diagram for recruitment into study and inclusion in the analytic sample (ie, rural adults ages 50+ years in the Northeastern United States).

This study is a secondary analysis of an existing survey dataset, and full details regarding recruitment and data collection are described elsewhere.^12^ In brief, participants were recruited (February-August 2021) using a Qualtrics Survey Panel, a research cohort selected from a larger pool of over 90 million adult volunteers.^13^ Eligibility criteria included: age 50+ years; resident of one of the following states in the Northeastern United States: Delaware, Maryland, New Jersey, New York, Ohio, Pennsylvania, West Virginia; and fluency in written English. Recruitment goals prioritized enrolling participants from rural counties and racial/ethnic minority subgroups. Potential participants were invited via email to complete a 30-min online survey. Informed consent was obtained from each participant by presenting the summary explanation of research before survey initiation. Qualtrics disseminated approximately 102 000 survey invitations, of whom, 2966 were eligible, enrolled, and provided complete data (Figure 1).

The analytic sample for the current analysis was limited to rural participants (n = 1052). Rural participants were identified based on the USDA’s rural-urban continuum code classification system via participants’ county of residence.^14^ Counties with rural-urban continuum codes ranging from 4 to 9 were considered rural for the purposes of this study.

The main study variables assessed were participant-reported demographics, travel time to healthcare, use of preventive healthcare, and health outcomes. Demographic information included gender, race, ethnicity, income, and type of health insurance. Travel time to healthcare was assessed with a single item: “On a typical day, how long, in minutes, would it take you to drive from your home to your doctor’s office?” Use of preventive healthcare was measured with questions about having a personal provider (ie, a “doctor, nurse, or other health professional that you see most often,” *no* or *yes*), time since most recent checkup (*within the past year* or *more than 1* *year ago*), and cancer screening behavior (as appropriate: up-to-date with colorectal, breast, and cervical cancer screening (*no* or *yes* for each item), and ever prostate cancer screening (*never* or *ever*)), using items drawn from national survey instruments.^15,16^ Questions about breast and cervical cancer screening were only asked of participants who identified as female, and prostate cancer screening of those who identified as male. Health status was measured using the *12-item short form* (SF-12) health survey (potential range: 0-100 for the physical health and mental health scores), and comorbidities were assessed with the *Charlson Comorbidity Index* (potential range of weighted scores: 0-25).^17,18^

Descriptive statistics were used to summarize mean travel time to healthcare, overall and by participant demographics. The relationship between study outcomes and travel time was expressed per 10 min traveled for all statistical analyses; that is, we assessed travel time in minutes continuously, but effect estimates are presented per 10 min to increase their interpretability. We used bivariate logistic regression models to assess the associations between travel time and dichotomous measures of preventive healthcare use, and we used multivariable logistic regression to repeat these analyses, controlling for demographic variables (age, gender, race/ethnicity, income, and insurance status). Similarly, we used bivariate and multivariable linear regression models to assess the associations between travel time and continuous measures of health outcomes, and bivariate and multivariable Poisson regression to assess the association between travel time and comorbidities score. Supplementary analyses examined the multivariable relationships between travel time and dichotomous responses to individual items from the Charlson comorbidity index, controlling for demographic variables.

All statistical analyses were conducted using SAS version 9.4 (Cary, NC). Analyses used a 2-sided *P*-value of .05.

Study methods were approved by the Penn State College of Medicine Institutional Review Board and Human Subjects Protection Office (protocol number 16024).

## Results

1052 rural adults participated in the study, and their demographic characteristics are outlined in Table 1. Overall, the mean travel time to healthcare was 18.5 min (standard error [SE] = 0.4; range: 0-60). Over half the participants were female (61%), non-Hispanic white (89%), and without private insurance (62%). Participants traveled significantly longer for healthcare if they reported higher income (*P* < .05) or a race other than non-Hispanic white (*P* < .01).

**Table 1.** Travel Time by Demographic Characteristics of Rural Residents.

	*n*	%	Mean travel (min)	SE	*P* value
Total	1052	100	18.5	0.4	
Age					.51
<65 years	465	44.5	18.5	0.6	
65+ years	579	55.0	18.4	0.5	
Gender					.73
Male	410	39.1	18.5	0.6	
Female	639	60.7	18.6	0.5	
Race/ethnicity					<.01*
Non-Hispanic White	938	89.2	18.4	0.4	
Other	114	10.8	19.7	1.4	
Annual household income					.02*
<$50 000	565	53.8	18.3	0.6	
$50 000+	485	46.1	18.8	0.5	
Insurance status					.19
Private insurance	397	38.2	19.1	0.6	
Other	643	61.1	18.3	0.5	

* Denotes statistical significance, with *P* ≤ .05.

Most participants reported timely use of preventive healthcare services: more than half had a personal provider, had a recent checkup, and were up-to-date with appropriate cancer screening (prevalence from 58% to 78%; Table 2). In bivariate and multivariable analyses, there were no significant associations between travel time and use of preventive healthcare.

**Table 2.** Prevalence and Odds of Preventive Healthcare Use Among Rural Adults Based on Travel Time, per 10 min Traveled.

	Prevalence	Bivariate		Multivariable	
		OR	95% CI	OR	95% CI
Have a personal provider	0.74	1.02	(0.91-1.13)	1.02	(0.91-1.14)
Recent check up	0.78	0.98	(0.88-1.10)	0.96	(0.86-1.09)
UTD CRC screening	0.63	1.05	(0.95-1.16)	1.06	(0.96-1.18)
UTD breast cancer screening	0.78	0.95	(0.81-1.12)	0.93	(0.79-1.10)
UTD cervical cancer screening	0.58	1.05	(0.86-1.29)	1.02	(0.83-1.26)
History of prostate cancer screening	0.66	1.11	(0.93-1.32)	1.19	(0.98-1.44)

Abbreviations: CI, confidence interval; OR, odds ratio; UTD, up-to-date.

Multivariable analysis controlled for possible confounding variables including income, sex, age, race, and insurance status.

Participants had moderate scores on the physical and mental health scales (physical health: mean = 45.4, SE *=* 0.65; mental health: mean = 50.4, SE = 0.7), with an average score on the Charlson comorbidity index of 1.6 (SE = 0.18; Table 3). In bivariate analysis, travel time was negatively associated with mental health scores and positively associated with scores on the comorbidity index. In multivariable analysis, greater travel time was associated with lower mental health scores (beta = -.62 per additional 10 min traveled, SE = 0.29, *p* = .03) and higher comorbidity index scores (beta = .11, SE = 0.02, *P* < .01), after adjusting for participant demographics. There was no relationship between travel time and physical health scores.

**Table 3.** Mean Scores and Associations for Health Outcomes Among Rural Adults Based on Travel Time, per 10 min Traveled.

	Mean score	*SE*	Bivariate			Multivariable		
			Beta	*SE*	*p*	Beta	*SE*	*p*
Physical health^a^	45.4	0.65	−.38	0.29	.18	−.36	0.29	.22
Mental health^a^	50.4	0.70	−.57	0.29	.05*	−.62	0.29	.03*
Charlson comorbidity index^b^	1.6	0.18	4.87	0.43	<.01*	.11	0.02	<.01*

Abbreviatios: SE, standard error.

Multivariable analysis controlled for possible confounding variables including income, sex, age, race, and insurance status.

a Assessed with linear regression models.

b Assessed with Poisson regression models.

* Denotes statistical significance, with *P* ≤ .05.

In our supplementary analysis, travel time was associated with several comorbidities included in the Charlson comorbidity index (Table 4). Specifically, in the multivariable models, greater travel time was associated with increased odds of having cancer (OR = 1.25 [95% CI = 1.05-1.48]) and diabetes (OR = 1.13 [95% CI = 1.01-1.27]).

**Table 4.** Prevalence and Odds of Comorbidities Among Rural Adults Based on Travel Time, per 10 Min Traveled.

	Prevalence	Bivariate		Multivariable	
		OR	95% CI	OR	95% CI
COPD	0.18	1.01	(1.00-1.02)	1.11	(0.98-1.26)
Arthritis	0.44	0.99	(0.99-1.01)	0.96	(0.87-1.07)
Cancer	0.07	1.02	(1.01-1.04)*	1.25	(1.05-1.48)*
Diabetes	0.21	1.01	(1.00-1.02)	1.13	(1.01-1.27)*
Digestive	0.12	1.01	(0.99-1.02)	1.10	(0.95-1.28)
Heart disease	0.14	1.01	(1.00-1.02)	1.09	(0.95-1.25)
HIV	0.02	1.01	(0.98-1.05)	1.15	(0.84-1.58)
Kidney disease	0.05	1.02	(1.00-1.04)	1.20	(0.99-1.50)
Liver disease	0.02	1.02	(0.99-1.05)	1.23	(0.92-1.64)
Stroke	0.04	1.01	(0.99-1.03)	1.16	(0.93-1.45)

Abbreviations: CI, confidence interval; COPD, chronic obstructive pulmonary disease; HIV, human immunodeficiency virus; OR, odds ratio.

Multivariable analysis controlled for possible confounding variables including income, sex, age, race, and insurance status.

* Denotes statistical significance, with *P* ≤ .05.

## Discussion

Our study utilized survey data to characterize the extent and implications of intrarural variations in rural patients’ travel time to healthcare. The study population consisted of older adults from rural counties in selected states of the Northeastern US, who had a mean travel time to healthcare of approximately 20 min. Travel time to obtain healthcare varied significantly by race/ethnicity and income. Interestingly, travel time was not associated with use of preventive healthcare services but was significantly associated with select health outcomes, including mental health and comorbidity scores. Our findings offer insight into the literature regarding the relationship between healthcare utilization and travel burden. Greater travel time among patients with poor mental health and higher comorbidity index scores may be related to a shortage of healthcare providers in rural communities or healthcare provider selection.

Certain demographic characteristics were significantly associated with travel time within our study sample. Rural adults with incomes greater than $50 000 a year had significantly higher travel times to healthcare. Previous studies analyzing healthcare access across various medical specialties have similarly found that higher income patients are less likely to obtain healthcare from the facility closest to them, and they are more likely to travel longer distances for healthcare.^19^ In addition, participants from minority racial/ethnic groups had higher travel times, which could be related to poor provider availability within traditionally marginalized communities (perhaps rural, predominantly minoritized communities). Previous research has shown that black Americans have higher travel burden to obtain healthcare, which may be a consequence of inequitable resource distribution and could contribute to racial/ethnic health care disparities in rural areas.^20^ Further research is needed to help understand the interaction of patient- and community-level factors, such as race, income, and resource distribution within rural communities, and their influence on travel time to obtain healthcare.

For rural older adults in our sample, greater travel time to healthcare was associated with poorer mental health scores. This relationship may reflect the shortage of mental health providers and resources in rural America, which results in increased travel time to obtain care.^21,22^ The connection between poor mental health and travel burden is supported by previous studies that found greater distance traveled to medical care is associated with fewer mental health visits and a decreased likelihood of receiving guideline-based mental healthcare.^23-25^ Rural patients are less likely than urban patients to receive mental healthcare.^26,27^ This difference may be caused by lower availability of services in rural communities.^28^ There is also evidence to suggest that mental health stigma in small rural communities contributes to a delay in patients seeking psychiatric care,^29,30^ which may result in poorer mental health outcomes. Although rural communities may be characterized by values, for example, stoicism and stigma that inhibit seeking mental healthcare,^31^ if mental healthcare services are available and accessible, rural patients will use them.^32^ These findings have policy implications and highlight an opportunity to advance equity in mental health by increasing geographic access to mental healthcare services in rural communities and providing support for patients who may face stigma.

Unlike mental health, physical health scores were not significantly associated with travel time. The SF-12 survey items used to assess physical health take into account factors including self-reported general health, physical functioning, perceived limitations, and pain.^18^ In contrast to the diagnoses captured by Charlson Comorbidity Index, these dimensions of physical health captured by the SF-12 physical health measure are more subjective, which could explain the lack of association between physical health and travel time. This difference between subjective and more objective measures of physical health among rural patients has been described by previous studies.^33,34^

In our supplementary analysis of comorbidity diagnoses, we identified 2 comorbidities that were more common among rural participants traveling greater distances to seek care: cancer and diabetes. These findings were not surprising since access to specialty care is generally poor in rural areas and consequently patients with certain diagnoses (eg, cancer) must drive farther to obtain more specialized care.^4,5,35^ Importantly, the measure of travel time we assessed in this study did not specify the type/specialty of doctor the participants traveled to; as a result, healthy participants without significant comorbidities may have answered the question in reference to accessing a primary care provider, whereas a patient with diabetes or cancer may have answered the question in reference to a more specialized provider (eg, a nephrologist or oncologist). However, patients with diabetes or cancer often still attend primary care. The travel time to these different types of providers will vary by availability and accessibility.

We expected the relationships between travel time and health to be related to differences in use of preventive healthcare services. Conceptually, decreased access to adequate prevention and/or treatment as a result of long travel times could result in greater comorbidities and generally poorer health. However, our study found no significant association between travel time and use of preventive healthcare services, including cancer screenings. Ability to detect an association with travel time may have been limited by the high prevalence level of participation in preventive healthcare, but it should be noted that uptake was about equal to national estimates (eg, up-to-date with breast cancer screening: 78% in our sample, compared to 76% reported in Healthy People 2030).^36^ These findings resonate with previous literature that has identified that access to transportation, not travel time or distance, impacts preventive healthcare utilization among rural populations.^8^

Overall, our study findings have implications for public health and healthcare policy. Our finding of a negative relationship between travel time and mental health suggests that the availability of mental health providers and services is lacking. Innovative interventions and policies are needed,^27^ such as increasing accessibility of telehealth for mental healthcare,^37^ expanding the mental health workforce,^28^ and leveraging the positive impacts of social support in rural communities.^38^ Our findings highlight a need for systematic changes that increase geographic access to both mental health and specialty care (eg, oncology and cancer survivorship services) in rural areas. Our results may be related to the rural hospital closures over the past decade, as there have been 136 rural hospital closures, nationally, between 2010 and 2021.^39^ Indeed, previous studies have connected rural hospital closures to increased travel distance and reduced access to healthcare.^6,40^ These closures disproportionately impacted patients who already had poor access to care including those with low income, lack of insurance, and minority race or ethnicity.^6,40,41^ Future research is need to further clarify the connection between hospital closures, patient travel time, healthcare utilization, and health outcomes among rural communities.

Our study’s strengths include the use of high-quality measures to assess constructs of interest with a relatively large sample size. In addition, the analysis of intrarural disparities is novel and can provide insight into the emergence of disparities and poor health outcomes in rural communities than direct rural/urban comparisons. Study limitations include limited generalizability given that our study population was comprised of a convenience sample of rural, predominantly non-Hispanic white, older adults from the Northeastern region of the US with access to and familiarity with online surveys; findings may not generalize to other settings or populations. In addition, we assessed travel time as minutes it would take to drive from the home to the doctor’s office, but some participants may typically travel for healthcare via other modalities (eg, with family, friends, or public transportation) or from other locations (eg, work). A related limitation is that the absolute differences in travel time were relatively small, which may limit their clinical significance. It is likely that this travel time varies based for patients with multiple doctors in different specialties or practices; however, our study only assessed 1 reported travel time. Future studies are needed to assess the reported relationships between travel time and health outcomes in different (rural) samples and with more comprehensive measures of travel burden and type of healthcare service accessed. In addition, future studies may consider different measurement and analysis approaches, including geospatial data collection and correction for multiple comparisons. Finally, the cross-sectional nature of our study precludes temporal inferences. Longitudinal studies would be helpful to determine how travel time gives rise to differences in health outcomes.

## Conclusions

Our study addresses a critical gap in the literature by highlighting the impact of intrarural travel time differences among older adults. We found differences in travel time to healthcare based on income and race/ethnicity. Increased travel time among rural older adults was associated with poorer mental health scores and more comorbidities (including cancer and diabetes) but was not associated with physical health scores or preventive healthcare utilization. Further research is needed to analyze how increased travel time might contribute to intrarural health disparities, as this relationship has likely been exacerbated by the amount of rural hospital closures over the last decade.
